# Super high-flux CVVHD using regional citrate anticoagulation: long-term stability of middle molecule clearance

**DOI:** 10.1186/cc14381

**Published:** 2015-03-16

**Authors:** M Siebeck, D Kindgen-Milles

**Affiliations:** 1 University Hospital, Düsseldorf, Germany

## Introduction

Conventional membranes used for CRRT have a limited middle molecule clearance. New membranes called super high-flux (SHF) or high cutoff membranes have been investigated. The loss of albumin with hemofiltration is a major drawback, but these membranes can be used in CVVHD with regional citrate anticoagulation (Ci-Ca® CVVHD), which may limit albumin loss, and contribute to a prolonged filter patency and an improved and stable middle molecule clearance. We evaluated saturation coefficients (SC), plasma clearances (PCL) and serum levels of eight small and middle molecules during 72 hours of Ci-Ca® CVVHD with a SHF membrane (Ultraflux®EMiC®2).

## Methods

After approval of the local committee of medical ethics and written informed consent we enrolled patients on a surgical ICU with AKI RIFLE-F who were treated with a Ci-Ca® CVVHD with a SHF membrane for 72 hours. We measured urea (0.006 kDa), creatinine (0.113 kDa), osteocalcin (5.8 kDa), B2MG (12 kDa), myoglobin (17.2 kDa), FreeLightChains (FLC) kappa (25 kDa) and lambda (50 kDa) and albumin (66 kDa) at 0 hours, 1 hour, 6 hours, 12 hours, 24 hours, 48 hours, and 72 hours. PCL, SC and serum levels during 72 hours were compared, using the Wilcoxon signed-rank test with *P *< 0.05.

## Results

Four females and 10 males (mean age 68.1 ±15.1 years; mean APACHE II score 13.7 ± 14.7; mean SAPS II 38.7 ± 12.7) were included. The SC and the PCL (ml/minute) of small solutes like creatinine at 1 hour (1.0 ± 0.0/23.72 ± 1.04) and 72 hours (0.95 ± 0.16/22.19 ± 3.99) were not statistically significantly different (*P *= 0.5/*P *= 0.42), the PCL was slightly reduced by 6%. The creatinine serum level was reduced by 42%. The SC and PCL of B2MG from 1 hour (0.61 ± 0.09/14.49 ± 2.5) to 72 hours (0.48 ± 0.13/11.6 ± 2.96) were significantly decreased (*P *= 0.0024/*P *= 0.0061). The reduction was 23% only; the overall clearance still was high. There was almost no reduction in SC or PCL for FLC kappa from 1 hour (0.176 ± 0.047/4.14 ± 1.08) to 72 hours (0.164 ± 0.078/3.854 ± 1.87), not reaching statistical significance (*P *= 0.94/*P *= 0.81). The serum levels of B2MG and FLC kappa were decreased by 39% and 23%. The SC of albumin was low (1 hour: 0.0009 ± 0.0004) and clearance decreased rapidly within the first 6 hours from 0.021 ± 0.01 to 0.011 ± 0.009. Serum levels of albumin did not decrease (1 hour: 2.64 ± 0.51; 72 hours: 2.63 ± 0.25).

## Conclusion

This study shows high middle molecular clearances using a SHF membrane with Ci-Ca® CVVHD for 72 hours with no loss of albumin. This set-up may improve blood purification in critically ill patients with acute kidney injury.

